# Concurrency of splenomegaly and numerous enlarged mesenteric and retroperitoneal lymph nodes in a patient with pelvic inflammatory disease caused by *Edwardsiella tarda*: Mimicking lymphoma

**DOI:** 10.1002/kjm2.12023

**Published:** 2019-03-19

**Authors:** Chien‐Hsiang Tai, Shu‐Fang Kuo, Chen‐Hsiang Lee

**Affiliations:** ^1^ Division of Infectious Diseases Department of Internal Medicine, Kaohsiung Chang Gung Memorial Hospital Kaohsiung Taiwan; ^2^ Department of Laboratory Medicine Kaohsiung Chang Gung Memorial Hospital Kaohsiung Taiwan; ^3^ College of Medicine Chang Gung University Kaohsiung Taiwan

Dear Editor,

1

*Edwardsiella tarda* is known to cause bacteremia and abscess. However, little is known about *E. tarda* septicemia with concurrent splenomegaly and lymphadenopathy, which mimics lymphoma.

A 45‐year‐old woman, who is a hepatitis C carrier, with type 2 diabetes mellitus and uterine leiomyoma, presented to our emergency department with a 3‐day history of fever, right lower quadrant abdominal pain, diarrhea, nausea, and vomiting. She had dietary with raw fish/sushi 2 days before this admission. Her right lower quadrant abdomen was noticeably tender with a palpable lymph node in the right inguinal area. Initial laboratory analysis revealed a white blood cell count of 20.9 × 10^3^/μL with 7.5% band cells. Abdominal computed tomography (CT) disclosed dilated uterine cavity with heterogeneous appearance and splenomegaly with numerous enlarged mesenteric and retroperitoneal lymph nodes (Figure 1A). Empirical antibiotic therapy with ertapenem was prescribed. Splenomegaly and numerous lymphadenopathies were noted in this febrile patient; lymph node biopsy was prescribed to exclude lymphoma.^1^

**Figure 1:**
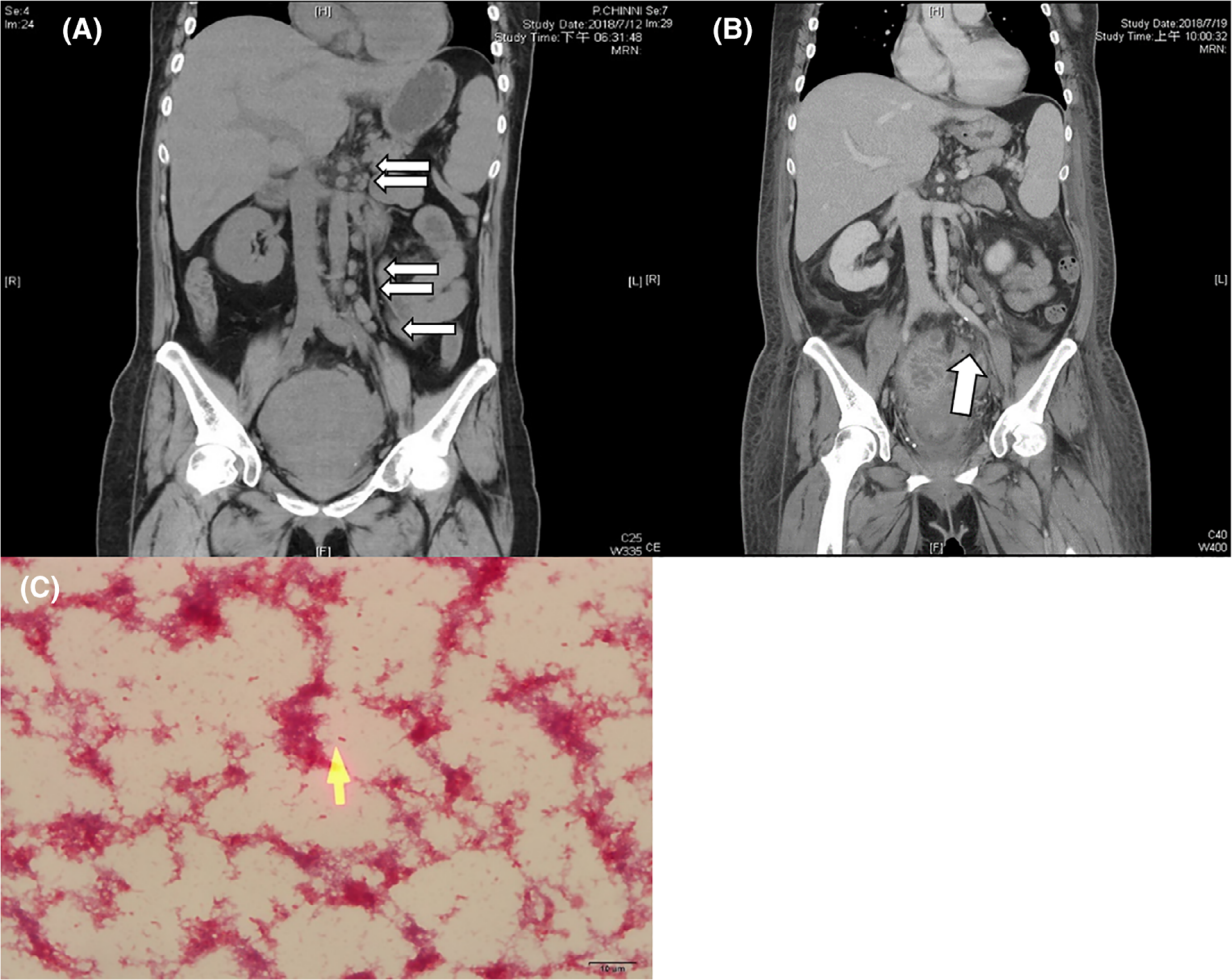
A, Initial abdominal computed tomography (CT) disclosed numerous enlarged lymph nodes (arrows). B, Repeated CT revealed rupture of uterus over the left posterior lateral wall (arrow). C, The Gram stain revealed Gram‐negative rod‐shaped bacilli (arrow) (original magnification, ×1000)

Two days later, the blood cultures yielded Gram‐negative rod‐shaped bacteria (Figure 1C). Identification tests by MALDI Biotyper system (Microflex LT; Bruker Daltonik GmbH, Bremen, Germany) revealed *E. tarda*, which was confirmed by the VITEK 2 Compact analyzer (bioMérieux, Marcy‐l'Étoile, France). Based on susceptibility test results, ceftriaxone therapy was administered to the patient. On the seventh day of admission, she underwent a right inguinal lymph node excision. The pathological report revealed lymphoid hyperplasia but not malignancy. The lymph node culture was not obtained.

Due to persistent fever, repeated CT revealed uterus rupture over the left posterior lateral wall (Figure 1B). On the 11th day, she underwent total abdominal hysterectomy and left salpingo‐oophorectomy. Ceftriaxone treatment was continued; the symptoms gradually improved. She was discharged on the 20th day. Follow‐up CT revealed the resolution of splenomegaly and lymphadenopathy.

*Edwardsiella tarda*, a Gram‐negative anaerobic rod that is not part of normal human flora, is found in fresh and brackish water environments and in many aquatic animals including reptiles, amphibians, and fish. *Edwardsiella tarda* infection is considered to be transmitted to humans by ingestion of contaminated food.^2^ *Edwardsiella tarda* is primarily associated with gastrointestinal diseases with manifestations of abdominal pain, diarrhea, nausea, and vomiting.^3^ Extra‐intestinal infections are relatively uncommon and include septicemia, salpingitis, tubo‐ovarian abscess,^4^ and pyomyoma.^3^ In previous studies, the major underlying diseases in patients with *E. tarda* bacteremia were liver cirrhosis, malignancy, diabetes mellitus, and hemochromatosis states.^2^ To the best of our knowledge, the concurrent incidence of splenomegaly and numerous lymphadenopathies in patients with pelvic inflammatory disease caused by *E. tarda* has not been reported. A study on the West African lungfish reported the presence of Gram‐negative rod‐shaped bacilli in the spleen, which had a DNA sequence identical to that of *E. tarda*.^5^ Further investigation is needed to determine whether the manifestations of *E. tarda* infection in humans are the same as that in lungfish.

In conclusion, we describe a case of *E. tarda* bacteremia with pelvic inflammatory disease presented with splenomegaly and numerous lymphadenopathies, mimicking lymphoma. It might help clinicians further recognize the various presentations of this organism.

## CONFLICTS OF INTEREST

All authors declare no conflicts of interests.
